# Exploring a long distance, amagmatic, across-suture orogenic geothermal system: Sri Lanka’s foreland hot springs

**DOI:** 10.1016/j.isci.2025.112370

**Published:** 2025-04-08

**Authors:** Dilshan Bandara, Jeroen Smit, Stefan Wohnlich, Thomas Heinze

**Affiliations:** 1 Institute of Geology, Mineralogy and Geophysics, Ruhr-University Bochum, Universitätsstraße 150, 44801 Bochum, Germany

**Keywords:** Earth sciences, Geology, Structural geology, Geophysics, Heat flow, Geothermal gradient

## Abstract

Hot springs in orogenic geothermal systems are usually within 5–15 km of the recharge zone, either within the mountain range or along the mountain front. However, in Sri Lanka, hot springs are in the foreland up to 100 km away from their recharge zones in the Highland Complex. The absence of a sedimentary cover provides the opportunity to study fluid pathways along basement faults and fractures. We identify a fracture network that connects the recharge and discharge zones oriented 015°–090°. Its orientation to the regional stress field indicates that the majority of the faults and fractures are permeable to allow fluid transport. With a geothermal gradient of ∼20°C/km obtained from 1D modeling, the estimated maximum circulation depth of the hot spring water is 3.5–5 km. Such foreland geothermal systems may also occur in other parts of the world hidden under a sedimentary cover, which could provide an immense geothermal resource.

## Highlights

• Thermal springs in Sri Lanka are orogenic with comparable circulation depths
• A 100 km long fracture network connects recharge zone and foreland hot springs
• Background heat flow sufficient for maximum reservoir temperatures

## Graphical abstract

**Figure:**
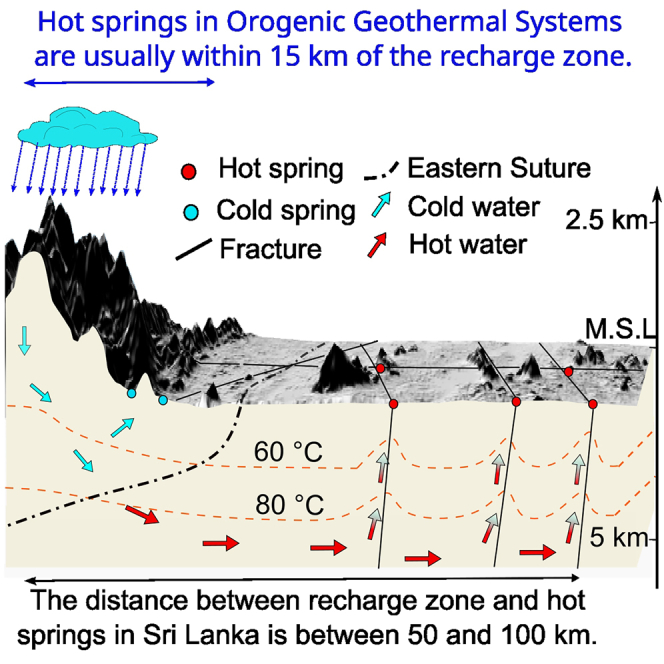

## Introduction

For the transition toward sustainable and renewable energy resources, geothermal heat is a corner piece. Understanding the mass and heat transport in thermo-hydraulic systems around the world is challenging because of their individual geological setting. However, due to these differences, common features might be more prominent in one system than in others and point to otherwise easily overlooked resources. This is the case for Sri Lanka, an old and non-magmatic tectonic setting. As such, Sri Lanka is not the typical location for thermal springs, but still nine hot springs are known in the eastern lowlands of the island. In this work, we investigate the idea that the geothermal system of Sri Lanka is in fact an orogenic geothermal system (OGS) and its far-reaching fracture network might also be present in other OGSs. This would provide substantial amounts of geothermal energy at previously disregarded locations.

**Figure 1:**
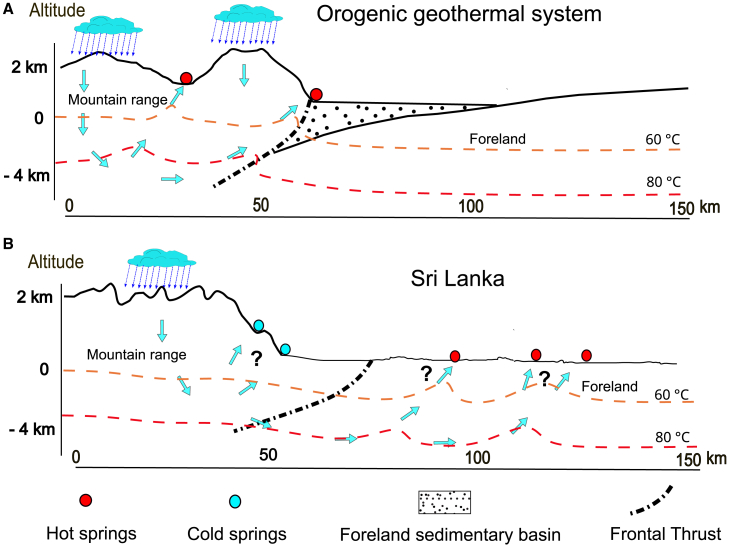
Concept of the study Classical orogenic geothermal system (OGS) vs. geothermal system in Sri Lanka. (A) Hot springs located within the mountain range and along a frontal thrust. (B) All hot springs located in the foreland, ca. 100 km from recharge zone. In the absence of a sedimentary cover, regional groundwater circulates through the crystalline basement in Sri Lanka. Not to scale.

In an OGS, the circulating water of meteoric origin is recharged in mountainous areas (orogen) and the fluid circulation is topographically driven by a high relief through deep-reaching permeable faults and fractures toward the discharge zones.^1^^,^^2^^,^^3^ The “orogen” and the fracture network of a system neither need to be formed simultaneously nor by the same tectonic processes to consider it an OGS. In fact, an active OGS may be related to an ancient orogen that possibly formed hundreds of millions of years ago, as in the case of Sri Lanka. Steeply dipping to vertical damage zones, such as faults and fractures form the fluid pathways. In a typical OGS, hot springs are located within 5–15 km from their recharge zone within the mountain belt itself or along the frontal thrust that acts as a natural boundary (Figure 1A).^4^^,^^5^^,^^6^ Spring temperatures can vary between 20°C and 100°C with peak reservoir temperatures of 100°C–250°C.^7^ Based on these temperatures and predicted geothermal gradients, the water reaches reservoir temperatures at an estimated maximum circulation depth of up to 10 km.^5^^,^^8^^,^^9^^,^^10^ Examples of OGS can be found in North America,^11^^,^^12^ the French Pyrenees,^13^ the Swiss Alps,^14^ the Himalayas,^15^ the Qilian Mountains, western China,^4^ New Zealand’s Southern Alps^16^ and Taiwan.^17^

Permeability heterogeneity and anisotropy of fault zones play a key role in such regional hydrogeological systems.^18^ Fractures and subsidiary faults act as fluid conduits, while fault cores commonly act as fluid flow barriers.^19^^,^^20^ Highly permeable fault zones facilitate a relatively fast upward flow, minimizing heat loss.^4^^,^^14^^,^^21^^,^^22^ In particular, (geothermal) springs are found in fault intersection zones due to the elevated permeability.^23^^,^^24^ The topographic difference between recharge and upwelling zones creates the topographic head, which drives deep fluid circulation. Additionally, temperature-dependent viscosity and density contrast between cold descending and hot ascending water promote self-channeling of the water.^25^ In Cenozoic mountain belts the foreland is covered with sediments, obscuring basement faults that may transport thermal waters tens of kilometers away from the mountain front, as is the case for the southern Alps foreland basin.^26^

**Figure 2:**
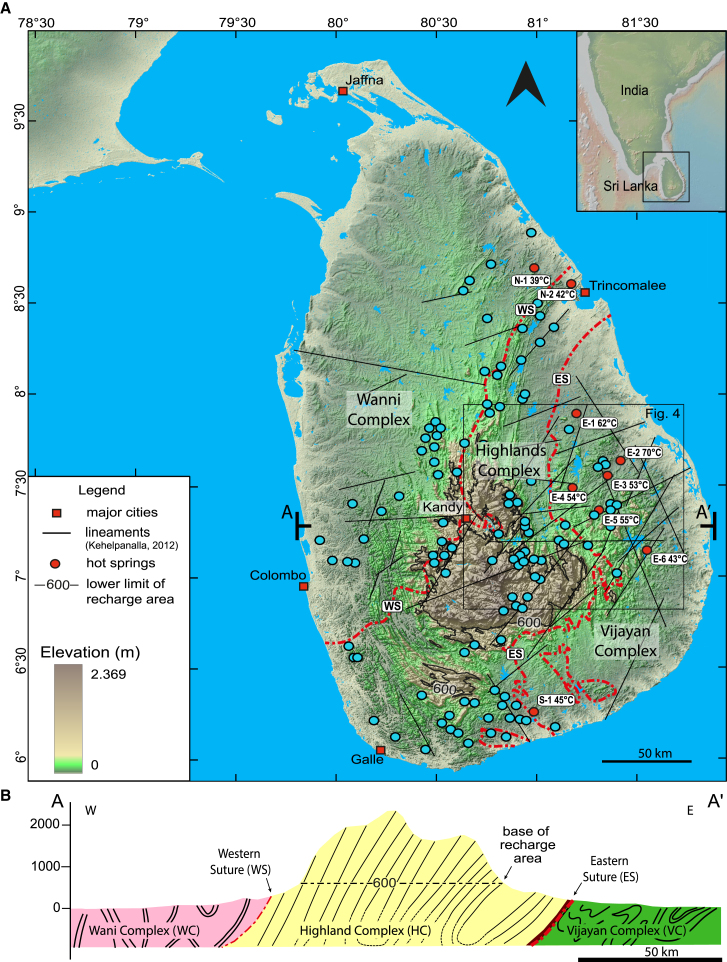
Geological map (A) General geological and lithotectonic map showing major fault and fracture zones, and hot (red dots—taken from Chandrajith et al.^27^) and cold (blue dots—taken from Panabokke^28^) springs. Hot springs E−1 to E−6 in the eastern foreland are the focus of this study. In the absence of a sedimentary cover, regional groundwater flows through faults and fractures in the high-grade metamorphic basement. Vijayan (VC) and Wanni (WC) complexes, arc-related crust, Highland Complex (HC) high-grade metasediments of subduction channel constitute the metamorphic basement that reached HP/HT, lower crustal conditions during the Pan-African Orogeny (ca. 600 Ma) (map modified after Cooray^29^; Kehelpannala^30^). Red lines indicate the Western and Eastern Sutures, and the black line marks the 600 m topographic contour line. Lineaments, thought to represent faults and fractures from satellite interpretation, as proposed by Kehelpanalla^30^ Inset: Location map of Sri Lanka in relation to India. (B) Coast-to-coast cross-section showing topographic variation in relation to lithotectonic units and basement geology (Chandrajith, 2020).^31^

In Sri Lanka, six of the nine hot springs are located in the orogenic foreland (E−1 to E−6 in Figure 2A), beyond the frontal thrust, at an exceptionally large distance of 50–100 km from the recharge zone.^27^ While the geothermal springs of Sri Lanka share most key features of a typical OGS (low temperature, meteoric origin recharged at elevated altitudes, maximum circulation temperatures of 100°C–120°C),^27^ their location in the orogenic foreland and the large distance to the recharge zone sets them apart from the aforementioned examples (Figure 1B). The frontal thrust of a mountain range can not only act as a fluid flow barrier but also as a conduit or an anisotropic mixture of both.^10^^,^^32^ In Sri Lanka, the frontal thrust is a, in places two km wide, Precambrian mylonitic zone. Therefore, together with the low permeable metamorphic rocks and the absence of a sedimentary cover, thermal water must circulate through weak zones in the crystalline basement (e.g., faults and fractures) into the foreland.^33^^,^^34^ The absence of a sedimentary cover in Sri Lanka provides the unique opportunity to study long-distance, across-suture fluid transport through basement weak zones.

### General geology of Sri Lanka

Sri Lanka is a fragment of a Precambrian active margin that can be linked to Mozambique, Antarctica, and India.^35^^,^^36^^,^^37^ Sri Lanka’s upper crust consists of three tectonic units composed of Precambrian high-grade metamorphic rocks (Figure 2B). From west to east: the Wanni (WC), Highland, and Vijayan Complexes.^38^ The WC and Vijayan (VC) complexes are two Precambrian magma arcs, while the Highland Complex (HC) consists of a high-grade metamorphic remnant of an accretionary complex (Figures 1B and 2).^39^

The Eastern Suture (ES) (or HC/VC boundary) is well-defined by up to ∼2 km wide (mylonitic) shear zones.^40^ The crust of Sri Lanka has undergone exhumation since the Ordovician^41^ and brittle deformation in different phases since at least the formation of the Indian Ocean approximately 160 Ma B.P.^41^^,^^42^ The latest major thermo-tectonic perturbation dates from the late Cretaceous and was related to opening of the Mannar and Cauvery basins, between Sri-Lanka and India.^43^ NNW-SSE compression in the Indian Ocean results from collision between India and Eurasia. Neogene acceleration along the Carlsberg Ridge around since ca. 8 Ma contributes to the NE-SW stress field in Sri Lanka and southern India^44^^,^^45^ and may have contributed to the present-day fracture network in Sri Lanka. Elements of this fault and fracture network can be traced over 100 km from the HC to the eastern coastal plains, crosscutting the ES (Figure 2A). Reportedly, these faults and fracture corridors have a width of up to 600 m.^41^

The upper crust in Sri Lanka consists of exhumed lower crustal rocks, including migmatites, granites, gneisses, and, mainly in HC, high-grade meta-sediments.^29^ As the primary porosity and permeability are negligible in these kinds of crystalline rocks, the deep, regional ground water flow mainly takes place along structural discontinuities like faults and fractures, foliation and bedding planes.^27^^,^^46^^,^^47^^,^^48^^,^^49^

### Present-day topography of Sri Lanka

Outside the HC, Sri Lanka experiences little altitude variations with maximum elevations between the sea level and altitudes of up to 270 m, also called the lowlands. On the contrary, the HC (Figure 2) incorporates elevations of up to 2,054 m.^50^ Opening of the basins between India and Sri Lanka during the late Cretaceous forms the last major thermo-tectonic perturbation, more than the ca. 40 Ma needed for a complete thermal reequilibration of the lithosphere. Despite this, the topography of the island is young and seems to be still evolving, as evidenced by the steep slopes and deep river incisions,^50^^,^^51^ potentially in response to ongoing geodynamics.

### Hydrogeological setting in the study area

Published hydrogeochemical parameters of hot spring waters are shown in Table S1. Hot springs in Sri Lanka have been categorized into three groups based on their major element concentrations: Na-Cl-HCO3 type (S-1, in the south of the island), Na-Cl-SO4 type (E−1, E−2, E−3, and E−5 in the eastern part of the island) and Ca-Cl-SO4 type (NE-1 and NE-2 in the north-eastern part of the island).^27^ Noticeably, the pH, electric conductivity, and major ion concentrations of hot spring water in the same terrain show similar values.^27^ Measurements of oxygen and hydrogen isotope ratios were used in Priyadarshanee et al.^52^ to identify the recharge areas of the thermal springs to be at “higher elevation”.

Out of the nine hot springs in Sri Lanka, two springs (NE-1 and NE-2 in Figure 2) are fed by locally precipitated meteoric water and have a travel time from recharge to discharge of less than 50 years as indicated by the presence of Tritium and the isotope ratios.^52^ The focus of this study is on foreland springs recharged at elevated altitudes. Hot spring S-1 is located along the ES, not in the orogenic foreland. Hence, this spring is also explicit from the current study.

Several heat sources have been proposed to explain the thermal springs, including magmatic intrusions,^53^ radioactive ore deposits,^54^ and an elevated geothermal gradient.^27^ The reservoir temperatures of the hot springs in the eastern foreland, calculated using silica and Na-Li geothermometers, range from 97° (E−5) to 120°C (E−1).^27^ Outflow temperatures range from 35° (E−6) to 72°C (E−3)^27^^,^^54^

The present study aims at understanding the geothermal system of Sri Lanka from the recharge zone in the Central HC to the discharge zone in the eastern lowlands (Figure 2). Specifically, we demonstrate that there is a long-ranging fracture network connecting recharge and discharge areas facilitating flow driven by a topographically induced hydraulic head. We apply a 3-fold approach consisting of (1) constraining the recharge altitude using stable isotopes, (2) constraining the maximum circulation depth by estimating the geothermal gradient using 1D modeling, and (3) identifying the fluid pathways by lineament mapping using satellite-based remote sensing and field observations. Once we defined the recharge area based on the topography of the island and the calculated recharge altitudes, we estimated the maximum circulation depth based on the calculated geothermal gradient and the maximum peak temperatures known from Chandrajith et al.^27^ Subsequently, with now well-defined recharge and discharge areas, we studied the fracture network connecting both regions and investigated whether the faults and fractures might be permeable to facilitate fluid flow. Combining our findings we present a conceptual model of the hydrothermal system in Sri Lanka.

## Results

### Constraining the recharge altitude from 600 to 1,200 m

The recharge altitude was calculated between 600 and 1,200 m for the geothermal water from the studied springs (Table 1). These altitudes are only present within the HC of the island (Figure 2). In general, the obtained isotope values may result from mixing of waters from different altitudes in a three-dimensional landscape along the fluids pathways. This would all result in an underestimation of the recharge altitudes.

**Table 1:** Recharge altitudes

Hot spring	δ18O (‰)	δ^2^H(‰)	Recharge altitude h (m)			
			Equation 1	Equation 2	Mean	Error
E−1^a^	−5	−30	540	730	630	100
E−1^b^	−5.5	−30.1	780	740	760	20
E−2^a^	−5.9	−36	970	1180	1070	100
E−3^a^	−6.3	−37	1160	1250	1200	40
E−3^b^	−5.5	−30.8	780	790	780	6
E−5^a^	−5.7	−35	870	1100	990	110
E−5^b^	−5.9	−33.2	970	970	970	1

Recharge altitudes calculated from the stable water isotopes using Equations 1 and 2 (Tsuchihara et al.).^55^ Hot springs names refer to Figure 2A.

a Chandrajith et al.^27^

b Priyadarshanee et al.^52^

Compared to the measurement accuracy of the provided isotope data, the discrepancy of the oxygen and hydrogen altitude relationships provided by Tsuchihara et al.^55^ has the strongest influence on the uncertainty estimation. Using a range-based uncertainty estimation we obtain an uncertainty of ∼50 m for the altitude estimation. Calculated values were rounded to tens of meters. The recharge altitudes calculated for the isotope data provided by Chandrajith et al.^27^ and Priyadarshanee et al.^52^ agree well within their range of uncertainty except for spring E−3. While similar methods were used in both studies, potential contamination during sampling or analytical errors cannot be excluded to explain this discrepancy.

Considering the calculated recharge zone altitudes of ∼600–1,200 m and the 40 to 100 m altitudes of the hot springs, the topographic head (recharge to discharge elevation difference) is in the range of 500–1,100 m (Figure 2). These topographic heads are consistent with values reported for other topographically controlled orogenic geothermal systems, such as ∼500 m in the Grimsel Pass in the Swiss Alps^5^ and ∼1,000 m in the French Pyrenees.^13^

### 1D modeling results in a geothermal gradient of 20°C/km

Calculations based on the parameters described in the Methodology section yield a geothermal gradient of 20 ± 1°C/km. A shallower Lithosphere-Asthenosphere boundary (LAB) (110 km) and Moho (30 km), with upper (18 km) and lower (12 km) crustal thicknesses yield a geothermal gradient of 21°C/km (case 1). A slightly deeper LAB (130 km) and Moho (40 km) and thicker upper (27 km) and lower crust (13 km) yields a geothermal gradient of 19°C/km (case 2).

Our modeled geothermal gradient of 20 ± 1°C/km agrees with those reported for Neoproterozoic crusts elsewhere, e.g., southern granulite belt in India, 12°C–19°C/km^56^ and Namaqualand, South Africa, 17°C/km.^57^ Higher radiogenic heat production of metasedimentary rocks, especially in the HC, explains the higher geothermal gradient calculated for Sri Lanka. The heat flow map by Lucazeau^58^ specifies the heat flow for Sri Lanka varying between 45 mW/m^2^ in the HC and 58 mW/m^2^ in the foreland. Using Fourier ’s law and mean thermal conductivity of rocks of 2.6 W/m/°C, the calculated geothermal gradients are around 17.3°C/km and 21.9°C/km. Hence, there is also a good agreement between the calculated geothermal gradient and global heat flow data.

Naturally, lateral variation of the geothermal gradient can be expected along the fluid pathways due to various influencing factors, such as topography, fluid circulation, and geological heterogeneity.^9^^,^^59^ Such variations become less pronounced with depth as microclimatic and topographic effects vanish toward greater depths.^60^ Due to infiltration of rainwater in the recharge area, the higher altitude, and the fluid circulation toward the hot springs, a shallower geothermal gradient can be expected beneath the recharge area,^9^ while hot water upwelling at the thermal springs might cause an increased geothermal gradient in the discharge areas.^61^

### The maximum circulation depth is 3–5 km

Based on published estimates of the reservoir temperatures of 97°C–120°C,^27^ our modeled geothermal gradient of 20 ± 1°C/km yields a maximum circulation depth of 3.5–5 km. An imaginary high geothermal gradient of ∼25°C/km would result in a maximum circulation depth of ∼2–3 km, an imaginary low geothermal gradient of 15°C–16°C/km would result in depths of 5–6 km. These values are well below the reported maximum circulation depths of 9–10 km estimated for the Grimsel Pass in the Swiss Alps.^5^ A circulation depth of 3.5–5 km is in line with the calculated 3.7 km for the Tet Fault in France^13^ and 1–5 km in North America.^11^ Similar values are also reported for other locations in the North Canadian Rocky Mountains, Taiwan orogens, and in New Zealand.^12^^,^^16^^,^^17^ All described hot springs in orogenic settings are driven by a topography-induced hydraulic head similar to the geothermal system in Sri Lanka. In summary, the estimated geothermal gradient of 20 ± 1°C/km alone can explain the reported peak circulation temperature of 97°C–120°C. This excludes the need for additional heat sources. In the following, we will investigate the presence of potential structures providing fluid pathways toward depth.

### Identified fracture network connects recharge and discharge zones

The lineament mapping focused on the area from the central HC (recharge zones) to the eastern lowlands (discharge zones) (Figure 4). Due to the absence of a stratigraphic aquifer, the lineament network is the only potential flow path (Figure 2B).

**Figure 3:**
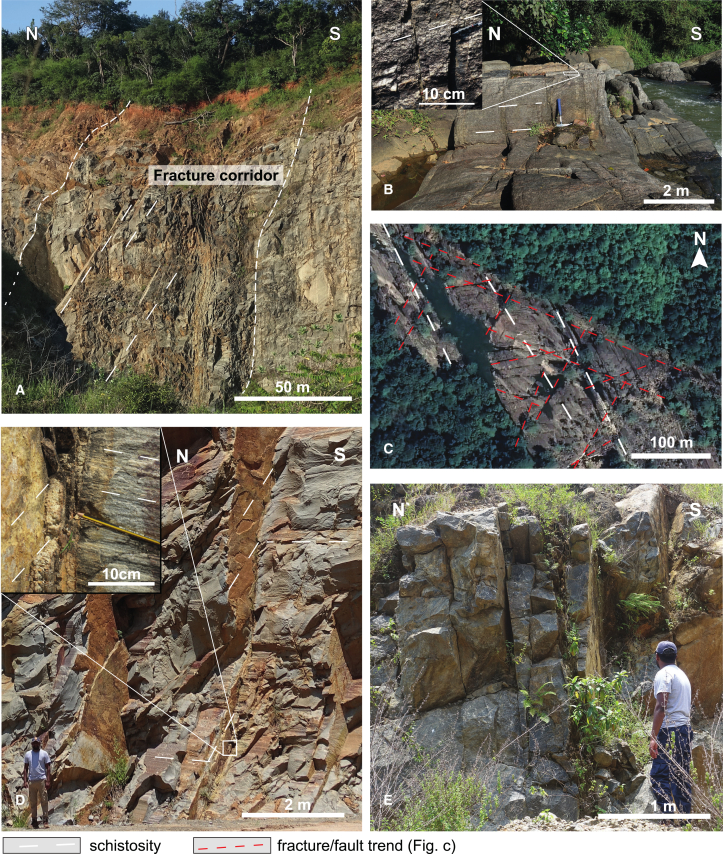
Outcrop examples (A) ∼100 m wide near-vertical fracture corridor in abandoned quarry near Moragahakanda dam. Fracture corridors may be regarded as incipient fault zones.^62^ (B) Near-vertical fault network in riverbed, with detail (inset), showing small >15° angles between fractures, as seen at regional scale (Figure 4). (C) Satellite image of fault-and-fracture network in bed of Mahaweli river near to Randenigala dam, not measured/analyzed in the field so technically these are lineaments (Source: Google Earth, USGS, image © 2024 Airbus). (D) Example of mirror-like fractures with meter-scale spacing in working quarry, inset shows chemically cm-scale weathering zone along multiple closely spaced discrete fractures. (E) Example of a roadside outcrop, with regularly spaced, sub-vertical fractures. All structures observed in outcrop are described as fractures as no clear shear-sense indicators or displacements were observed. See Figure 4 for locations.

**Figure 4:**
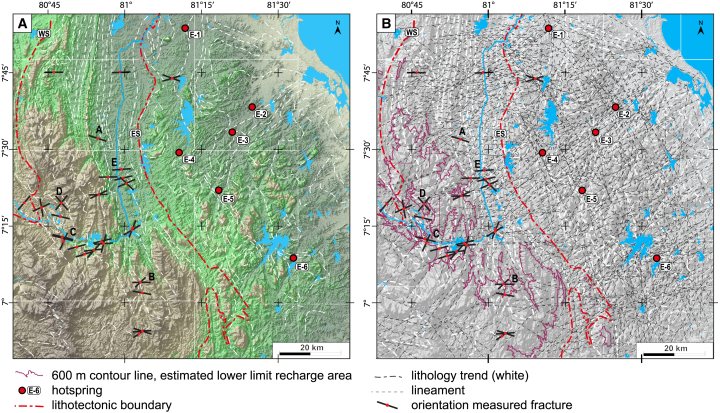
Fracture network identification (A) colored shaded-relief map (SRTM 1 arc second, NASA JPL^63^) of central and eastern Sri Lanka showing full extent of the studied geothermal system, from recharge to discharge zone, major lineaments are clearly visible. (B) Shaded relief map with network of all (628) mapped tectonic lineaments. Seemingly curved lineaments are composed of several straight segments, facilitated by the small angles between lineaments. Letters A–E refer to Figure 3.

**Figure 5:**
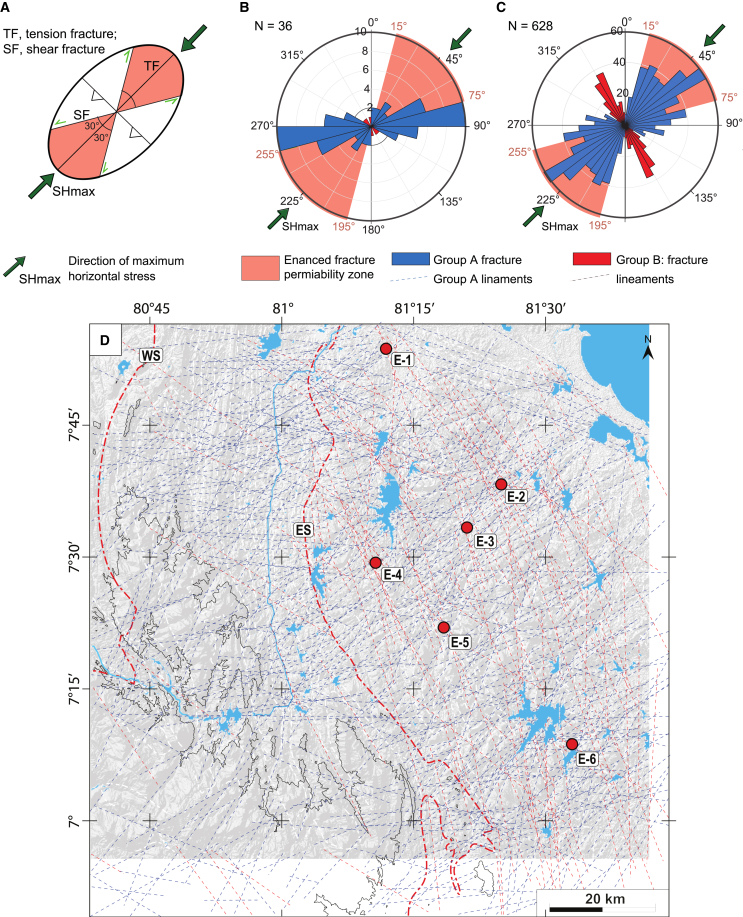
Fracture network analysis (A) Plane-strain ellipse with expected fracture orientations and zones of enhanced fracture permeability in an NE-SW-oriented compressional stress field, represented by maximum horizontal stress, SHmax. Fractures orthogonal to the NE-SW-oriented maximum compressional stress field are predicted to be closed. (modified after Rogers^67^ and Magee^68^). The NE-SW direction of the SHmax in Sri Lanka is based on local hydro-frac measurements (Pahlavan et al.)^69^ and the regional present-day stress field (Jade et al.^44^; Demets et al;^45^). (B) Rose diagram with the distribution of fractures measured in the field. n, number of elements plotted (C) Rose diagram with the distribution of lineament orientations from remote sensing (see Figure 4) and the expected zones of enhanced fracture permeability (red) in the present-day NE-SW stress field. Group A, 15°–90°, centered between ∼45°–60° (blue) and B, 120°–180°, centered at ∼150° (red), shows a strong concentration around 150° based on distribution. (D) Shaded relief map with lineament network color-coded according to lineament groups A and B.

The tectonic lineament analysis yields a pervasive network of lineaments with a higher density in the lowlands of the Vijayan complex than in the mountainous region of the HC. Seemingly curved lineaments are composed of several straight segments, facilitated by the small angles between lineaments. Two lineament clusters can be defined in the satellite-based dataset (Figures 4 and 5). The main cluster with the majority of the orientations between 15° and 90°, is centered between ∼45° and 60° (group A in Figure 5). A second, much smaller cluster with orientations between 120° and 180°, shows a strong concentration around 150° (group B in Figure 5). From Figure 4, it appears that group B lineaments are general in the east and largely absent in the HC, west of the ES. This can be explained by the structure and composition of the basement. The HC basement consists of metasediments, their layering and parallelism to the ES (Figure 4, white dashed lines) may have the following effects. The layering concentrates erosion (likely better than fractures), layer-parallel slip between layers may perform the role of fractures and fractures that do form will be harder to distinguish. On the contrary, the lithology in the VC exhibits a patchy, irregular and coarse pattern, markedly different from the straight tectonic lineaments. In total, our dataset contains 628 satellite-based structural lineaments (Figure 4) and 36 measured fractures (Figures 3 and 4). The main orientation of the fracture network, group A lineaments (015°–090°), can be traced from the central HC to the eastern coast and the hot spring fields. These crosscut the ES (Figure 4). The second, relatively small cluster forms a 120°–180° trending system that is prominent in the eastern lowlands of the VC. Most hot springs are located in the vicinity of high-angle intersections between the 015°–090° system and the 120°–180° fracture systems (Figure 4). This suggests that the smaller 120°–180° fracture system plays a key role in the upwelling of geothermal water and spring localization.^64^ The small fault offsets and image resolution do not allow recognition of cross-cutting relationships. The elements of the dataset cut the topography in straight lines, indicating they are sub-vertical (Figure 4). This sub vertical dipping of fractures is confirmed by our field observations (Figure 3).

Kehelpannala et al.^41^ reported field observations of sinistral displacement along an ∼ EW faults in the central HC. Although we didn’t find evidence for faulting, fractures are concentrated in 50–100 m wide zones of subparallel fractures with fracture densities of ∼0.5–50 m^−1^ a number of outcrops (e.g., Figures 3A and 3B). These fracture corridors may accumulate displacements with continued deformation and become brittle faults.^62^ As such, fracture corridors represent an intermediate stage between early distributed fracturing and discrete faults and may be regarded as incipient faults. In places, 10–30 cm wide zones of discolored, loose material may represent either fault gauge or severe alteration by water circulating through finely spaced fractures (Figure 3D, inset).

Regarding fracture orientations observed in the field and using satellite imagery, the discrepancy in sampling size needs to be considered before interpreting possible differences. In our case, outcrops are concentrated in a small part of the study area. A number of outcrops are located along one of the main roads from Kandy to the east close to the E-W fault described by Kehelpannala et al.^41^ Other reasons for such differences include a mismatch in observational scale and the fractures’ capability to localize erosion, which are the main criteria for visibility in satellite images. Based on the number of observation points, the field-based orientations are a small subset of the satellite-based observations.

### The regional stress field benefits across-suture fracture network as flow paths

Fractures parallel to the maximum horizontal stress axis (SHmax) and oblique to it within ±30° are likely to be open, and to have enhanced permeabilities due to tensile and shear stresses and are more favorable to fluid flow (Figure 5A).^65^^,^^66^ In contrast, fractures perpendicular to the direction of SHmax are likely to be closed due to the orthogonal stress field and are not conducive to fluid flow (Figure 5A).^67^^,^^68^^,^^70^^,^^71^^,^^72^

Hydrofrac stress measurements in 250–850 m deep boreholes drilled for geotechnical investigations revealed that the present-day maximum horizontal stress field (SHmax) is oriented NE, ∼045° in Sri Lanka.^69^ The recent GPS velocity measurements in the Indian plate are also in good accordance with the 045° SHmax orientations for the island.^44^^,^^45^ In addition, the results of numerical simulations of the present-day stress field in the Indian subcontinent also confirm the similar 045° orientation of the SHmax for Sri Lanka.^73^

Figure 5 shows the rose diagram of the mapped lineaments from remote sensing with the ±30° range of the present NE–SW SHmax of Sri Lanka highlighted. Most of the fractures in the study area are oriented within this range with respect to SHmax (015°–070° and 195°–255° zones shown in Figure 5). This suggests that these fractures are active, open, and permeable enough to transport fluid over such a long distance from recharge to discharge zones. The pervasive nature of the fracture network, its high fracture density (Figure 4) and the enhanced permeability due to SHmax (Figure 5) suggests that permeability is high and that the network is able to provide an effective conduit for recharged meteoric water from recharge to discharge areas and for 3.5 to 5 km deep penetrations that form the conduits for an unusual geothermal system.

The 120°–180° oriented fractures, as well as many parts of the ES, are perpendicular to the SHmax and may therefore be closed by orthogonal compression and have no or low permeability, thus acting as fluid flow barriers. Therefore, it can be argued that fluid flow along the enhanced permeability fractures (oriented 15°–90°) are blocked once they intersect the low permeability 120°–180° fractures and water begins to upwell along them. This argument is well supported, as most springs are located at or near the aforementioned fracture intersections (Figure 4). For the ES, several cross-cutting fractures oriented 15°–90° seem to overcome the potentially low hydraulic conductivity of the ES. However, the Mahaweli River might experience subsurface inflow along the ES, which have not been detected or studied so far, agreeing with the existence of springs at fracture intersections.

There is a very small number of seismic events recorded for Sri Lanka,^74^ which limits the gain of further insights into fracture reactivation and permeability. Reservoir induced seismicity recorded for the Victoria Dam in the Central HC indicates the failure of steeply dipping fault planes in NNE–SSW (085°) direction but not of faults oriented in NW–SE (128°) direction.^75^

In the Euganean Geothermal System (EuGS), NE Italy, meteoric water is recharged at ∼1,500 m above M.S.L. and circulates ∼70 km to the foreland basin through strike-slip faults.^26^ While in the EuGS water circulates through faults in sedimentary rocks, in Sri Lanka, water recharged at high altitudes (>600 m) circulates through strike slip faults in metamorphic rocks. The example from the EuGS shows that recharged meteoric water at the mountain range can circulate a large distance of ∼100 km through the secondary permeability of rocks, such as regional fracture zones.

### Fracture intersections might facilitate hot water upwelling

Hot springs are located at an intersection of two or more fractures (Figure 4D). A similar setting is reported from France by Taillefer et al.,^21^ where most springs are located near intersections of subsidiary faults with the Tet Fault. In addition, Curewitz and Karson^23^ also reported several worldwide examples of source localization in fault interaction zones and fault tips, at the Juan de Fuca Ridge (northeastern Pacific Ocean), Hengill (Iceland), the East African Rift (Kenya), the Menderes Massif (Turkey), and the Waiotapu geothermal field (New Zealand). The presumably higher permeability at fracture intersections shortens the ascending time, decreasing temperature loss, thus forming a hot water spring at the surface. Similar to other amagmatic orogenic geothermal systems where discharge sites are located at valley floors or along the coast,^7^ the springs in Sri Lanka are located at low elevations indicating the relevance of the hydraulic head gradient on the localization of the springs.^64^ However, the relationship between hydraulic head gradient and ascending time and temperature loss might by non-linear due to various factors, such as buoyancy, contributing, and counteracting water uprise.^7^

No further indication of hot water upwelling is found closer to the recharge area, along the mountain front, the ES or in elevated mountain valleys despite passing steep topography and high hydraulic head gradients (Figure 4). He et al.^39^ reported abundant mineralization along the ES and at least part of the alluvial plains located all along the mountain front and in the valleys contain impenetrable clay layers.^76^ Therefore, upward fluid flow along the fracture is impeded by confining mechanisms, i.e., mineralization and clay clogging, that can slow down the upwelling of water, thereby cooling the water prior to discharge.^77^ Such confining mechanisms can lead to an effectively impermeable layer trapping the fluids at depth.

Based on the presented analysis, yet unexplored geothermal resources are most likely to be found at fracture intersections along the fractures with enhanced permeability (015°–070°, Figure 5B) due to the NE-SW-oriented SHmax. This information narrows down the potential locations for future geothermal exploration.

## Discussion

**Figure 6:**
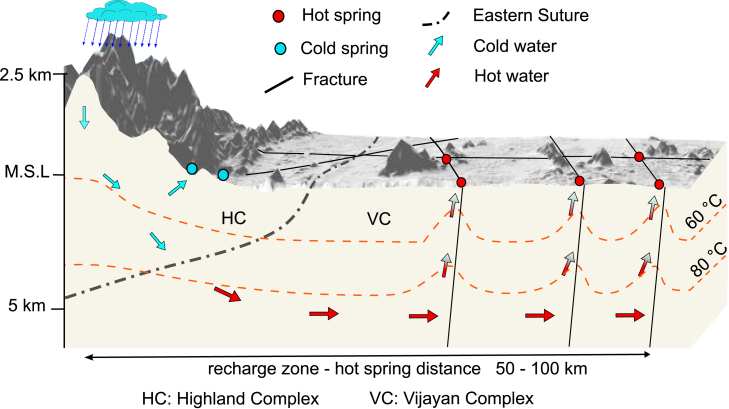
Conceptual model Hydrothermal fluid flow in geothermal system in Sri Lanka. View is along a potential near-vertical fracture plane in E-W direction. Meteoritic water is transported from high-elevation recharge zone to depth and across the frontal thrust into the foreland to emerge in hot springs. Note the importance of highly permeable fault zones conduit water from recharge to discharge, crossing the mountain front. Recharge area—hot springs distance is ca. 100 km compared to 5–15 km for classical OGS.

Based on our analysis we propose a general conceptual model for the formation of hot springs and for the geothermal system in Sri Lanka (Figure 6). As in classical OGS, meteoric water is recharged at elevated altitudes (>600 m) to infiltrate to a depth of up to 5 km, driven by a topographically induced hydraulic head. In Sri Lanka, geothermal water flows through permeable, cross-cutting fractures across the mountain front to form hot springs in the foreland. As a result, in Sri Lanka, hot springs are located in the orogenic foreland at distances of up to 100 km from the respective recharge zones. Like in the classical OGS, the recharged meteoric water is heated by the regional heat flow without additional local heat sources as demonstrated by our geothermal modeling. The reservoir temperature depends on the depth of fluid circulation as a function of the topographically induced hydraulic head and the availability of fluid pathways toward depth. Water flowing through longer faults and fractures surfaces at intersections with other fractures, forming hot springs.

Classical OGS’s are confined to the mountain range with hot springs located in the mountains or at its base along the frontal thrust commonly resulting in distances of roughly 5–15 km between springs and recharge zones, with very few exemptions such as the EuGS.^26^ The presence of a long-running, regional fracture network in Sri Lanka suggests that there are fluid pathways covering the distance contrary to classical OGS and that this deeply circulating water can cross the mountain front where highly permeable fracture zones dissect the frontal thrust/shear zone. The absence of a sedimentary cover allows the easy recognition of fluid flow paths by satellite remote sensing and of hot springs in the flat lowlands of Sri Lanka.

There are many similarities and good correlations between the EuGS of Italy and the long-distance geothermal system of Sri Lanka, such as topographic gradient and circulation depth. This suggests that similar long-distance geothermal systems may exist in different geological settings in other parts of the world. However, the circulating geothermal water may be obscured by the overlying sedimentary cover in many foreland basins, such as the EuGS, and only becomes clearly visible in Sri Lanka without such a sedimentary cover. In foreland basins with a sedimentary cover, mixing with shallow groundwater resources in the sedimentary cover and small discharge rates of the springs may further mask the geothermal system in the foreland. In such foreland basins, currently, geothermal exploration is limited to the mountain ranges or their mountain fronts, as geothermal activities are only reported in these regions. However, the present study results underline the potential availability of additional geothermal resources outside the mountain ranges and provide a new perspective for future geothermal energy exploration programs in foreland basins in general. Therefore, the identification of fracture networks that cross the mountain front and their potential continuation under the foreland sedimentary cover may help to discover hidden geothermal resources.

### Limitations of the study

This work presents the first systematic study of the fracture network in Sri Lanka spanning from the HC to the thermal springs in the lowlands. Few studies addressed fractures in Sri Lanka in the past and were based on a limited number of surface observations. Hence, there is little data available besides the piloting results obtained throughout this study and future work needs to address this knowledge gap through further investigations and ground truthing of the fracture network across the island mapping fracture orientations and dip.

Whereas the fracture network is prominent and recognizable from satellite imagery, field conditions can be challenging. Fault and fracture zones are abundant and readily visible in relatively fresh and unaltered outcrops. However, due to the high weathering rates under influence of the tropical climate, such outcrops are limited to active quarries, recent road cuts and certain riverbeds and therefore few and far between. Furthermore, the lack of calcite or other mineral deposits along the fractures and evidence of offset relationships in the massive, homogeneous crystalline host rocks, the field dataset does not allow for more advanced field kinematic analysis.^78^^,^^79^ As the focus of this work is on the identification of long-running, regional scale lineaments connecting recharge, and discharge areas of the geothermal waters, there is a detection bias due to the manual identification and scale considered. An in-depth analysis of the lineament network of the island will provide further insights into its structural composition. Such a characterization of the structural and geological environment of the geothermal system including its lithology could provide further information about potential fluid pathways. If further and deeper borehole logs become available, fracture density and aperture with depth could be analyzed to provide further insights into the depth-dependent hydraulic behavior of fracture flow. Especially the role of the ES as a potential hydraulic barrier or conduit needs to be investigated further to understand the passage of geothermal water across the mountain front in Sri Lanka contrary to classical OGS. Including the presence of cold-water springs and the role of the Mahaweli River, which might experience subsurface inflow along the ES.

The estimation of the maximum circulation depth depends on the modeled 1D geothermal gradient. Direct geothermal gradient measurements (e.g., through temperature logging at sufficiently deep boreholes) would improve the estimation of the geothermal gradient, and subsequent estimations of the maximum circulation depth, significantly. Age determination of the geothermal waters could also help to address uncertainties in the applicability of the used isotope relationships used to constrain the recharge zones.

## Resource availability

### Lead contact

Further information and requests should be directed to and will be fulfilled by the lead contact, Thomas Heinze (thomas.heinze@ruhr-uni-bochum.de).

### Materials availability

This study did not generate new unique reagents.

### Data and code availability

• SRTM imagery is available via the earthexplorer provided by USGS. All other data are directly available in the article itself and in referenced articles.
• No original code was used for the processing or plotting of data in this article. The geothermal gradient modeling was done via the open-source software B1T 1D. MATLAB and QGIS were used for data processing and visualization.
• Any additional information required to reanalyze the data reported in this article is available from the lead contact upon request.

## Acknowledgments

First author acknowledges the 10.13039/501100001655German Academic Exchange Service (DAAD) for funding the doctoral project.

## Author contributions

D.B. conducted the recharge altitude calculations. D.B. and J.S. conducted the geothermal gradient modeling, the lineament analysis and produced the figures. D.B., J.S., and T.H. analyzed and discussed the result, drafted the original and revised version of the manuscript. S.W. supervised the project and contributed to the editing of the article.

## Declaration of interests

The authors declare no competing interests.

## STAR★Methods

### Key resources table

**Table:** 

REAGENT or RESOURCE	SOURCE	IDENTIFIER
**Available data**		

Isotope data	27, 52	https://doi.org/10.1016/j.hydrol.2012.11.004 https://doi.org/10.1016/j.apgeochem.2021.105174
SRTM imagery	open data	https://earthexplorer.usgs.gov

**Software and algorithms**		

B1T 1D	open software	https://www.thermogis.nl/en/publications
MATLAB	MATLAB 2023b	https://de.mathworks.com
Qgis 3.18	open software	https://qgis.org/download/

### Method details

We apply a 3-fold approach in the study, including estimation of recharge altitudes of spring water using stable isotopes, estimation of geothermal gradient and maximum depth of groundwater circulation using 1D thermal modeling, and identification of the fracture network providing the fluid pathway using structural analysis based on remote sensing and field measurements.

#### Recharge altitude estimation

The ratio of stable isotopes is a common indicator to estimate recharge altitudes of water bodies as precipitation at higher altitudes is depleted in heavier isotopes. This so-called "altitude effect" provides a relationship between isotope ratios in precipitation and elevation. Groundwater recharged at higher altitudes will show a respective isotopic signature of precipitation from that elevation.^80^

Tsuchihara et al.^55^ derived a relationship of the altitude effect on stable water isotopes based on isotope values from streams, lakes, wells, springs, taps, and bottles collected along a transverse profile across Sri Lanka from the wet zone to the dry zone. We used the following two equations reported in Tsuchihara et al.^55^ to estimate the recharged altitudes of the geothermal waters:

(Equation 1) $$\delta^{18}O=-0.00211\cdot h-3.8669\text{,}$$

(Equation 2) $$\delta^{2}H=-0.0135\cdot h-20.1561\text{,}$$

where h is the recharged altitude of the geothermal spring water and δ^18^O and δ^2^H are measured isotope values from the discharged geothermal springs water.

The known isotope ratios of δ^18^O and δ^2^H for the geothermal spring waters reported by Chandrajith et al.^27^ and Priyadarshanee et al.^52^ were used in the equations provided in Tsuchihara et al.^55^ to estimate the geothermal water recharge altitudes. The water samples for the isotope analysis were processed and analyzed in an almost similar way with comparable analysis uncertainties.^27^^,^^52^ There was no isotope data available for hot springs E−4 and E−6. Both used literature sources (Chandrajith et al.^27^; Priyadarshanee et al; ^52^) provide isotope values for springs E−1, E−3 and E−5.

A similar method has been used in several studies to calculate recharge altitudes of meteoric water in orogenic geothermal systems using the local δ^18^O gradients with altitude.^5^^,^^13^

General drawback of the applied method is the unavailability of the paleoclimatic data and the water recharge ages for the area. However, the final glacial period reported in Sri Lanka has ended at least before 15,000 years^81^^,^^82^ Thus, we assumed that water recharge occurred recently (<15,000 years) and isotopic fractionation is similar to present day climatic enabling the application of the work of Tsuchihara et al.^55^ for the recharge altitude estimation of the geothermal spring waters. Uncertainties are estimated based on the discrepancies of the equations for oxygen and hydrogen and the measurement accuracy of the used isotope data.

#### Geothermal gradient modeling and circulation depths

The geothermal gradient was modeled for a first-order estimate to test whether background heat flow alone could heat the water to the reservoir temperatures (100°C–120°C) reported by Chandrajith et al.^27^ at a depth similar to reported OGS (<10 km). We used the B1T 1D temperature and rheological model^83^ that provides a solution of the steady-state conduction heat equation. The temperatures at the surface and at the Lithosphere-Asthenosphere boundary (LAB) are upper and lower boundary conditions. The temperature at the LAB is commonly set to 1300°C marking the brittle to ductile transition.^83^^,^^84^ Comparably shallow heat flow in the upper crust cannot be considered independent of heat flow properties at greater depth. Hence, considering heat flow and generation down to the LAB, e.g., tributes to the influence of the radiogenic crust and stress dependent thermal parameters in a heterogeneous subsurface. Thermal parameters and heat sources are specified for three model layers (in absence of a sedimentary cover), namely upper crust, lower crust and lithospheric mantle (see Table S1). The vertical resolution is 100 m.

The depth of the LAB below Sri Lanka is estimated to vary between 110 and 130 km using gravity, magnetic, heat flow and seismic data in a lithospheric density model^84^^,^^85^^,^^86^ and the depth to the Moho between 30 and 40 km derived from the data of a temporary seismic network in 2016 & 2017.^87^ Parameters were chosen according to literature values.^58^^,^^76^^,^^88^^,^^89^^,^^90^^,^^91^ Together with the absence of a sedimentary cover, LAB and Moho depth are the most critical and uncertain parameters in our calculation of the geotherm. The calculated uncertainty is determined from the calculation of maxima and minima based on the uncertainty in LAB depth, depth to Moho and thicknesses of the upper and lower crusts (case 1 and case 2 in Table S1).

Maximum circulation depths of meteoric water were calculated for each known hot spring based on published estimated maximum reservoir temperatures,^27^ discharge temperatures of hot spring water and our modeled geothermal gradient. The maximum reservoir temperatures were calculated with silica based and cation based geothermometers.^27^ The temperature gain through the geothermal gradient is the difference between the peak circulation temperatures and the temperature of the infiltrated meteoric water, assuming that rainfall temperature and soil surface temperature is equal to the mean annual air temperature (25°C).

#### Fracture network mapping

To analyze the continuity and the permeability of a potential fault and fracture network from recharge to discharge areas, we used satellite remote sensing and field work for ground truthing. Mapping focused on the area from the central Highland Complex (recharge zones) to the eastern lowlands (discharge zones). By mode of remote sensing the network of tectonic lineaments was mapped. Tectonic lineaments are defined as rectilinear or curvilinear features in the landscape that are believed to represent the surface traces of faults and fractures.^62^^,^^92^ The tectonic lineament network was mapped from a shaded-relief map that we generated from 1-arc sec. Shuttle Radar Topography Mission (SRTM) imagery (https://earthexplorer.usgs.gov) with a 30 m horizontal resolution.^63^ Lineaments were drawn following elevational discontinuities or structural breaks were observed with elevation differences on the shaded-relief map with a scale of 1: 50,000. Azimuth angles of the shaded relief maps were varied during lineament identification to improve the visibility of structural breaks. The identified lineaments were subsequently compared with published literatures, satellite images and geological maps (e.g., Kehelpannala^30^; GSMB, 1:100,000 geology maps) to prevent the misinterpretation of lithological boundaries and schistosity as faults or fractures in the study area, as well as satellite images were used to exclude anthropogenic structures, such as (rail)roads.

Tectonic lineament may be referred to as a fault or fracture (corridor) after its nature is confirmed on the ground, so-called ‘ground thruthing’. Field investigation was conducted in fresh outcrops at 27 locations (Figure 4) to verify the nature of the mapped tectonic lineaments. Presumably, fault displacements are small and accommodated along multiple faults in wide zones, which complicates the distinction between fractures and faults. It is telling that scanning electron microscope imaging was used to demonstrate the presence of a cataclasite along the fault.^41^ We did not find clear indications for fault displacement in outcrops and therefore, all structures are described as fractures. The strike, dip, and density of the fractures were measured at each location. Due to local conditions, field verification is limited to a small number of outcrops that are concentrated in the Highland Complex (Figure 4). Outcrops include working and abandoned quarries, road outcrops and river beds (Figure 3). A few sections of the Mahaweli river provide the best and largest outcrops, some visible and mapable on satellite images (e.g., Google Earth, Figure 3C). Mainly in the dry seasons, the riverbed offers hectometer to km-scale outcrops for the study of fault-and-fracture network at intermediate scales.

The remote sensing analysis focused on the identification of continuous tectonic lineaments with a length of more than 5 km potentially connecting recharge and discharge zones. Considering the resolution of the used remote sensing data and geological maps, there is no claim in completeness for lineament identification. From the set focus of this study and the manual lineament analysis, there is a detection bias with respect to the number of lineaments potentially falsifying any quantitative interpretation of the overall network. However, the applied methodology in combination with ground truthing is sufficient to investigate the potential existence of a network connecting recharge and discharge zones of the thermal springs and to subsequently evaluate the effect of the local stress field on these identified lineaments. Shaded relief map generation, georeferencing and fracture analysis were carried out in QGIS. Rose diagrams representing the remote-sensing-derived lineament orientations were used for the quantitative analysis of the fault-and-fracture network (Figures 5B and 5C). The rose diagram was generated using MATLAB.

### Quantification and statistical analysis

There are no quantification or statistical analyses to include in this study.
