# Supplementary material for: Exploring a long distance, amagmatic, across-suture orogenic geothermal system: Sri Lanka’s foreland hot springs
Source: iScience. 2025 Apr 8;28(5):112370. doi: 10.1016/j.isci.2025.112370 (PMC12053636; doi:10.1016/j.isci.2025.112370)
Supplement: Document S1. Table S1 [file mmc1.pdf]

iScience, Volume *28*

Supplemental information

Exploring a long distance, amagmatic, across-suture orogenic geothermal system: Sri Lanka's foreland hot springs

Dilshan Bandara, Jeroen Smit, Stefan Wohnlich, and Thomas Heinze

Table S1: Model parameters, related to STAR Methods. Input parameters used in the geothermal gradient modeling with relevant references.

Name	Case 1	Case 2	Units	Reference
Thickness of the Lithosphere	110	130	km	Kumar et al. [60]
Depth to the Moho	30	40	km	Dreiling et al. [63]
Thickness of the Upper Crust	18	27	km	Dreiling et al. [63]
Thickness of the Lower Crust	12	13	km	Dreiling et al. [63]
Density of the Upper Crust	2730	2730	$g\ cm^{-3}$	Büchel[64]
Density of the Lower Crust	2900	2900	$g\ cm^{-3}$	Kumar et al. [60]
Density of the Mantle	3300	3300	$g\ cm^{-3}$	Kumar et al. [60]
Temperature of the LAB	1300	1300	°C	Kumar et al. [60]
Thermal conductivity of the Upper Crust	3.3	3.3	$W\ m^{-1}K^{-1}$	Schön[65]
Thermal conductivity of the Lower Crust	2.6	2.6	$W\ m^{-1}K^{-1}$	Schön[65]
Thermal conductivity of the Mantle	3	3	$Wm^{-1}\ K^{-1}$	Schön[65]
Mean surface temperature	25	25	°C	Mapa[66]
Radiogenic heat production of the Upper Crust	1.48	1.48	$\mu W\ m^{-3}$	Vila et al. [67]
Surface heat flow (data from southern granulite terrain in India)	45	45	$mW\ m^{-2}$	Lucazeau[68]
Radiogenic heat generation Upper Crust as percentage from surface heat	30%	30%	-	Hasterok & Chapman[69]
